# Alterations in cerebrospinal fluid levels of myelin- and oligodendrocyte-related proteins in sporadic Creutzfeldt–Jakob disease

**DOI:** 10.1186/s40478-026-02247-5

**Published:** 2026-02-10

**Authors:** Fabian Maass, Carolina Thomas, Lille Kurvits, Peter Hermann, Matthias Schmitz, Mathias Bähr, Christine Stadelmann, Sabrina Zechel, Sezgi Canaslan, Inga Zerr

**Affiliations:** 1 Department of Neurology, University Medical Center Göttingen, Robert-Koch-Str. 40, 37075 Göttingen, Germany; 2 Department of Neuropathology, University Medical Center Göttingen, Göttingen, Germany; 3 Centre for Neuropathology and Brain Research, Paul-Flechsig-Institute, Leipzig, Germany; 4 German Center for Neurodegenerative Diseases (DZNE), Göttingen, Germany; 5 National Reference Center for CJD Surveillance, University Medical Center Göttingen, Göttingen, Germany

**Keywords:** Creutzfeldt–Jakob disease, Cerebrospinal fluid, Biomarker, Myelin basic protein, NG2, CNPase

## Abstract

Recent evidence suggests glial dysfunction, particularly involving oligodendrocytes and myelin, as an important part of the primary disease mechanism of sporadic Creutzfeldt–Jakob disease. To the best of our knowledge, cerebrospinal fluid (CSF) biomarkers reflecting oligodendrocyte or myelin damage have not yet been systematically investigated in this context. CSF samples from patients with sporadic Creutzfeldt–Jakob disease (CJD), Alzheimer's disease (AD) and non-neurodegenerative controls (n = 18, respectively) were included in the study, and levels of myelin basic protein (MBP), neural/glial antigen 2 (NG2) and cyclic nucleotide 3′-phosphodiesterase (CNPase) were quantified. Additionally, p25-positive cells were quantified in autopsy tissue from the frontal and parietal cortical regions of five patients with CJD and four controls. In the primary cohort, MBP quantification revealed significantly higher CSF levels in CJD compared to AD (*p* = 0.004) and controls (*p* = 0.0001) and this difference remained significant after adjustment for age and neurofilament light chain (NfL), indicating that MBP elevations cannot be explained solely by neuroaxonal degeneration. Significant differences were also found for CNPase in CJD compared to AD (*p* = 0.0011) and controls (*p* = 0.0004). There were no differences in NG2 levels (*p* > 0.05) among the groups. However, no significant differences were observed in the total number of p25-positive mature oligodendrocytes between CJD and controls in cortical brain tissue (*p* > 0.05). Additionally, MBP and NfL were quantified in a second cohort of 28 CJD and 20 biomarker-defined AD patients. In this cohort, characterized by more advanced AD pathology, MBP strongly covaried with NfL and no longer differed between diagnostic groups, suggesting a greater influence of neuroaxonal injury on MBP levels in advanced disease stages. In conclusion, in CJD, MBP elevations appear to be only partly related to neuroaxonal degeneration and may reflect disease-related myelin involvement.

**Supplementary Information:**

The online version contains supplementary material available at 10.1186/s40478-026-02247-5.

## Introduction

Sporadic Creutzfeldt–Jakob disease (CJD), as the most prevalent prion disease in humans, is characterized by rapidly progressive neurodegeneration due to the accumulation of misfolded prion protein [1]. Typically regarded as a grey matter encephalopathy, recent evidence suggests glial dysfunction, particularly involving oligodendrocytes and myelin, as an important part of the primary disease mechanism. While white matter degeneration was initially believed to be a secondary consequence of neuronal loss, more recent studies suggest a primary involvement of oligodendrocytes and myelin in the pathophysiology of prion diseases [2–6]. Prion protein deposition in oligodendrocytes appears to be a rare finding and was mainly described in patients with the MM2C subtype and longer disease duration [7]. Therefore, other factors may contribute to myelin and oligodendrocyte damage. Recent GWAS and transcriptomic studies on CJD have revealed a set of regulations that are consistent with primary oligodendroglial dysfunction [3, 5]. Additionally, advanced diffusion MRI techniques revealed early and widespread microstructural alterations in major white matter tracts [2]. These findings raise the possibility that oligodendroglial and myelin pathology in CJD is not merely a consequence of neuronal damage but may constitute an autonomous pathological process. Besides CJD, oligodendroglial dysfunction has also been implicated as a contributing factor in several other neurodegenerative diseases [8].

Cerebrospinal fluid (CSF) analysis provides a valuable tool to investigate CNS disease mechanisms in vivo. Here, we present a quantitative analysis of myelin basic protein (MBP), 2′,3′-cyclic-nucleotide 3′-phosphodiesterase (CNPase) and neural-glial antigen 2 (NG2) in a CJD cohort enrolled at the German National Reference Center for CJD Surveillance in Göttingen, complemented by a second independent cohort in which MBP was quantified. For comparison, patients with Alzheimer’s disease (AD) and non-neurodegenerative controls were also included. This was complemented by post-mortem assessment of oligodendrocytes in brain tissue in a second independent cohort. AD was chosen as a disease control because it is also a progressive neurodegenerative disorder driven by protein misfolding and predominant grey matter pathology. This permits a more specific assessment of white matter changes attributable to CJD rather than to general grey matter–related neurodegeneration. Our results suggest the involvement of oligodendrocyte- and myelin-related pathology in CJD and highlight the potential diagnostic and mechanistic relevance of glia-derived CSF markers in prion disease.

## Materials and methods

### Study population (CSF analysis)

CJD and AD samples were obtained from the biobank of the German National Reference Center for Transmissible Spongiform Encephalopathies (NRZ-TSE), Göttingen, Germany and the biobank of the Dept. of Neurology, University Medical Center Göttingen, Göttingen, Germany. CJD cases were retrospectively selected based on two predefined criteria: definite sporadic CJD confirmed by post-mortem neuropathology and availability of residual CSF after completion of routine diagnostic testing. Clinical data from patients with CJD were collected in the context of a study of the NRZ-TSE on epidemiology and biomarkers for prion diseases. All CJD patients exhibited pathological Real-Time Quaking Induced Conversion (RT-QuIC) CSF results and matched neuropathological criteria for definite CJD [9]. AD samples for the primary cohort were collected in the context of a monocentric longitudinal study of rapidly progressive Alzheimer's disease (rpAD). Patients were enrolled from the dementia outpatient clinics of the University Medical Center Göttingen, Germany and the German National CJD Surveillance Unit between 2008 and 2021 and matched clinical criteria for AD [10] plus at least one biomarker criterion for AD-associated amyloid or tau pathology [11]. In this cohort, 11 of 18 AD cases were amyloid-positive (A+) based on a decreased CSF Aβ42/Aβ40 ratio, and two additional patients had neuropathologically confirmed AD pathology. The remaining amyloid-negative (A–) cases nonetheless exhibited a clinically typical AD phenotype, including characteristic neuropsychological deficits and supportive imaging findings. We also analyzed a second independent CSF cohort. Here, all AD patients (n = 20) fulfilled biomarker-defined amyloid positivity (A +), in line with current ATN recommendations [12]. Additionally, CSF samples from control subjects (CTRL) were selected who did not display any clinical signs of neurodegeneration but were comparable in age and gender characteristics. CSF amyloid beta-42, beta-40 and the amyloid beta 42/40 ratio were measured in all controls to exclude AD-related amyloid pathology. Selection criteria for both cohorts can be found in the supplement.

Ethical approval was obtained from the Ethics Committee of the University Medical Center Göttingen (Nr. 13/11/12, Nr. 37/11/21, Nr. 9/6/08, Nr. 11/11/93, Nr. 39/2/19). Written consent was obtained from all patients or caregivers. The study is in accordance with the Code of Ethics of the World Medical Association (Declaration of Helsinki).

### CSF sampling

CSF samples were collected according to international guidelines for standardization of cerebrospinal fluid biobanking [13]. Briefly, the first 2–4 ml of CSF were retained for routine analysis (WBC, RBC, total protein, total albumin), while subsequent CSF was collected in polypropylene tubes. Samples were then centrifuged, aliquoted and stored at − 80 °C.

### Analyses of CSF proteins

Amyloid β1-40, amyloid β1-42, total-tau and phospho-tau 181 levels were quantified using ELISA kits from Fujirebio (Fujirebio, Ghent, Belgium). These systems have been previously certified (CE marked) for use in the clinical setting and are currently used in our department. An in-house Real-time Quaking Induced Conversion (RT-QuIC) assay and a standard 14-3-3 ELISA test were used for CJD diagnostics, as described previously [14]. Commercial ELISA kits were used to quantify myelin basic protein (MBP), neural/glial antigen 2 (NG2) and cyclic nucleotide 3′-phosphodiesterase (CNPase) according to the manufacturers’ guidelines (MBP: AnshLabs, Texas, USA; NG2: Abcam, Cambridge, UK; CNPase: Biomatik, Ontario, Canada). Assay metrics can be found in the supplement. NfL concentrations were measured using the Lumipulse G Neurofilament Light immunoassay (Fujirebio, Japan) on the fully automated Lumipulse G600II platform, based on a chemiluminescent enzyme immunoassay (CLEIA) principle.

### Human brain tissue and TPPP/p25 immunostaining

Formalin-fixed, paraffin-embedded autopsy tissue from the frontal and parietal cortex of five patients with clinically suspected CJD and histopathological PrPSc deposition as well as four age-matched controls showing no/minimal pathological changes (NIA-AA Scores A0-1 B0) at routine brain autopsy were randomly selected for the study from the 2015–2020 archive of the Institute of Neuropathology at the University Medical Center Göttingen, Germany (see Supp. Table 1). Vacuolation and neuronal loss was absent in the control patients and raked from mild to severe in the CJD cohort (see supplementary Supp. Table 2). To determine the densities of mature oligodendrocytes, 3 µm tissue sections were incubated with an antibody against Tubulin Polymerization-Promoting Protein (TPPP /p25; Abcam, ab92305, 1:500) after heat pretreatment in Tris/EDTA buffer, ph 8.0, and antibody binding was developed using 3,3′-diaminobenzidine (DAB). Alzheimer’s disease–associated pathology was evaluated using Bielschowsky silver staining and immunohistochemical chromogenic staining with antibodies against β-amyloid (6E10, Zytomed) and tau (Pierce, Thermo Fisher) in all patients included in the histopathological cohort. A summary of the findings, scored according to Reiniger et al. [15], and the NIA–AA criteria [16] is provided in the Supplement.

### Oligodendrocyte quantification in post-mortem human brain tissue

Slides were digitized using an Olympus VS120 Scanner at 200 × magnification (pixel size: 0.35 × 0.35 µm). A QuPath project was created to select regions of interest (ROIs), and whole slide images (WSIs) were loaded into the software. Six rectangular ROIs per slide were selected from both the cortex and white matter (12 ROIs in total) and exported to ImageJ software using the Fiji open-source platform for biological-image analysis [17]. The exported RGB-ROIs were converted to 8-bit images and analyzed using a custom-made macro. Briefly, a representative background region of the image was manually selected. Using the following formula, a threshold was set to detect p25-positive cells: *Threshold* = *round*(*μ*_*background*_ − 7·*σ*_*background​*_). The obtained threshold was visually inspected for accuracy and manually adjusted if necessary. Using the selected threshold, particle analysis was performed in Fiji, including particles with a size range of 4–50 µ^2^ and circularity between 0.35 and 1.00. PrP deposits (positive for PrP 3F4—MAB1562, Millipore- and/or PrP 12F10 – Bertin Pharma Spibio A03221) were analyzed semiquantitatively (low-moderate vs. high) in the neighboring gray matter by a trained neuropathologist (CT; Supp. table 2).

### Statistical analysis

The distribution of the data was assessed both visually, using quantile–quantile (Q-Q) plots, and statistically, using the Shapiro–Wilk normality test. Qualitative variables were compared using the chi-squared test. To evaluate group differences of CNPase, NG2 and MBP, the Kruskal–Wallis test with Dunn’s correction for multiple comparisons was applied. Additionally, MBP levels were compared between groups using linear regression, adjusting for age and NfL levels. For the comparison of NfL values, Kruskal–Wallis test followed by uncorrected Dunn’s test was applied. Differences between two groups were assessed using the Mann–Whitney U test. Correlations between two variables were analyzed using Spearman’s rho. The 95% confidence interval (CI) for the area under the receiver operating characteristic curve (AUROC) was calculated using the Wilson/Brown method. All statistical analyses were conducted using GraphPad Prism version 9.4.1 or the R language 4.3.1.

## Results

### Study population

Characteristics of the study population of the primary cohort can be found in Table 1. There were neither significant difference in age nor sex between the three groups (*p* > 0.05). In the AD cohort, the median disease duration was two years, and the median Mini-Mental State Examination (MMSE) score was 23 points, indicating a less severely affected AD cohort.

**Table 1:** Characteristics of the study population in the primary cohort

	CJD	AD	CTRL
Patients (n)	18	18	18
Age (years)	66 (38)	72 (24)	62 (40)
Male/female	9/9	9/9	10/8
14-3-3 AU/ml	64,000 (107,840)	NA	NA
p-Tau pg/ml	56 (88)	73 (224)	NA
Tau pg/ml	NA	506 (1773)	NA
Amyloid-β 1–40 pg/ml	NA	11,266 (14,934)	NA
Amyloid-β 1–42 pg/ml	NA	745 (1140)	NA
Amyloid-β 1–42/1–40 ratio	NA	0.06 (0.08)	NA
Neurofilament light pg/ml	6512 (7296)	1596 (2947)	437 (1459)

Data is presented as median (range). CJD = Creutzfeldt–Jakob disease, AD = Alzheimer’s disease, CTRL = control subjects, NfL = neurofilament light chain, NA = not applicable

### Quantification of 2′,3′-Cyclic nucleotide 3′-phosphodiesterase (CNPase), myelin basic protein (MBP) and neural/glial antigen 2 (NG2) in the CSF

**Fig. 1:**
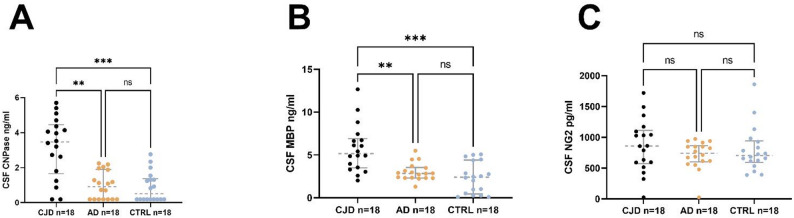
Quantification of CSF myelin basic protein (MBP), neural/glial antigen 2 (NG2) and 2′,3′-Cyclic nucleotide 3′-phosphodiesterase (CNPase) **A**, **B** Increased levels of CNPase and MBP can be detected in CJD compared to AD and controls. **C** No difference in NG2 levels could be detected between the different groups

In the control group, CNPase levels above the limit of quantification (LOQ) were detected in only 50% of cases (9 out of 18). In Alzheimer's disease, values above the LOQ were detected in 61% of cases (11 out of 18 patients). In the CJD group, values above the LOQ were detected in 89% (16 out of 18 patients). For further analysis, non-quantifiable samples were set at the LOQ to allow statistical analysis. Significant differences were found between CJD and AD (*p* = 0.0011) and CJD and CTRL (*p* = 0.0004). There was no difference between AD and CTRL (*p* > 0.05) (Fig. 1A).

**Fig. 2:**
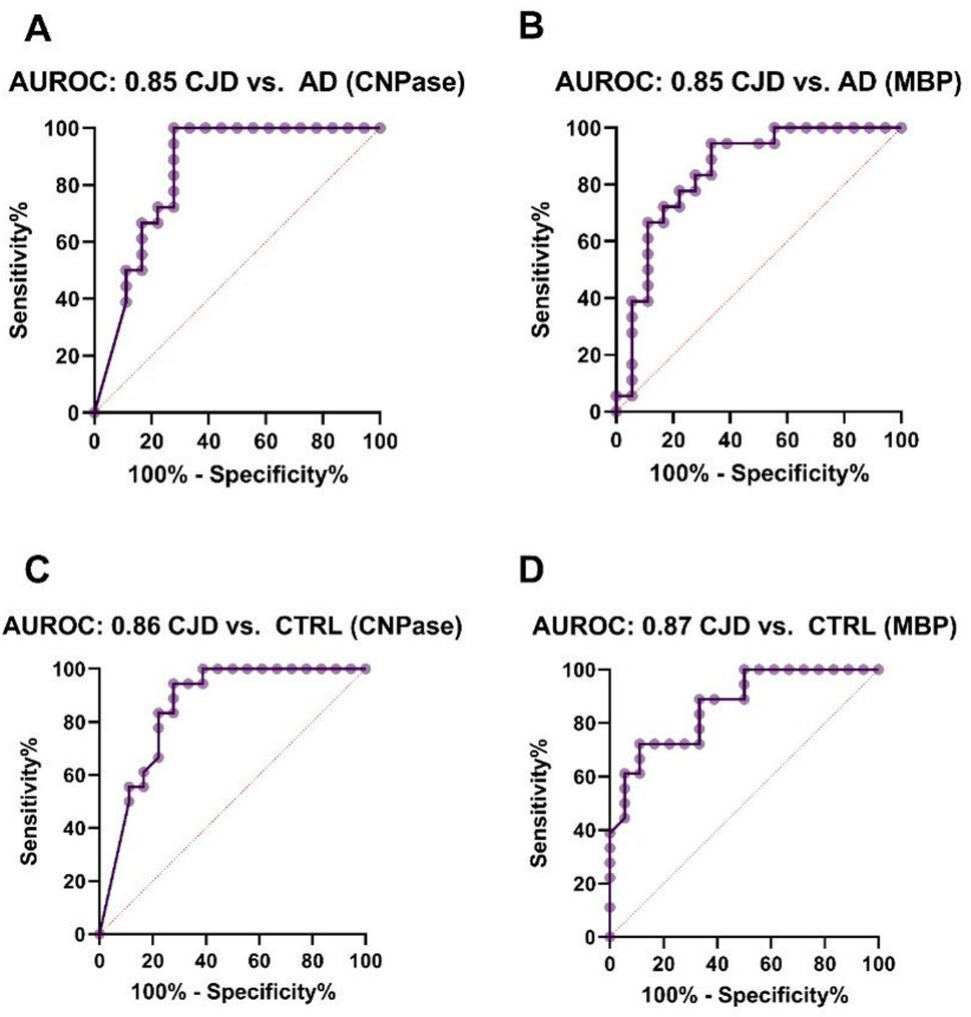
ROC curves for the discrimination of CJD, AD and CTRL applying CNPase and MBP CSF levels. Significant discrimination could be demonstrated for all comparisons

The results of the MBP analysis revealed significantly higher CSF levels in CJD compared to AD (*p* = 0.004) and compared to the control group (*p* = 0.0001), respectively (Fig. 2B). There was no difference between AD and CTRL (*p* > 0.05).

**Fig. 3:**
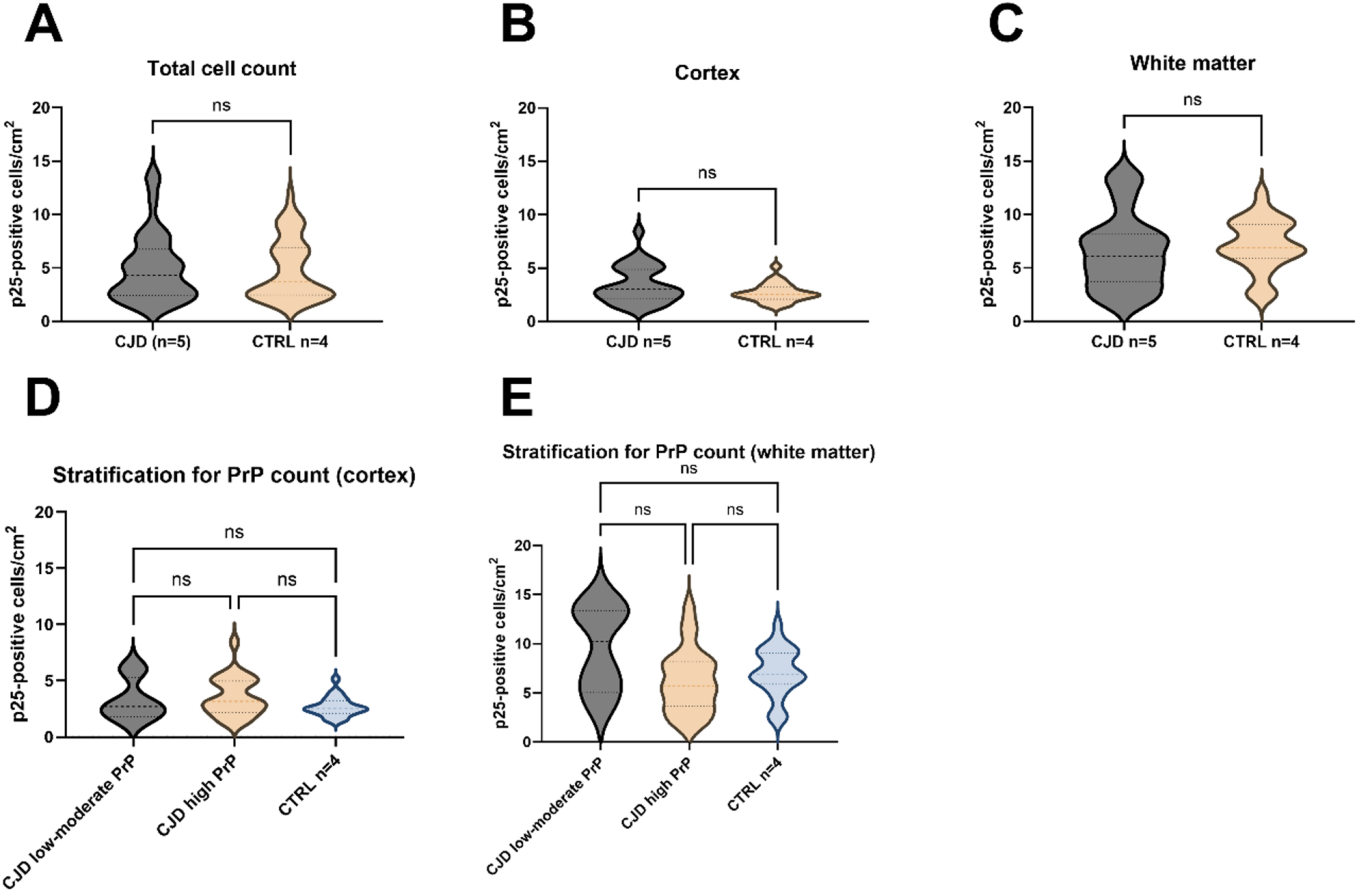
Oligodendrocyte quantification in CJD and control brain tissue. Stratification for white matter and cortical regions, as well as for high and low-moderate PrP count in the context of CJD, revealed no significant differences

No group differences were found for NG2 CSF quantification (*p* > 0.05) (Fig. 3C).

### ROC-analysis applying CNPase CSF levels

A ROC analysis was conducted to assess the discriminatory value of CSF CNPase and MBP levels. The analysis revealed significant discrimination between CJD and AD (CNPase: AUROC 0.85, 95% CI 0.71–0.99, *p* = 0.0004; MBP: AUROC 0.85, 95% CI 0.71–0.98, *p* = 0.0004) (Fig. 2A, B), as well as between CJD and controls (CNPase: AUROC 0.86, 95% CI 0.73–0.99, *p* = 0.0003; MBP: AUROC 0.87, 95% CI 0.75–0.98, *p* = 0.0002) (Fig. 2C, D).

### Correlation between CSF CNPase, MBP and NG2 levels and clinical data

No significant correlation was observed between age and the corresponding CSF levels of the three CSF markers after adjusting for multiple comparisons according to Bonferroni (adjusted significance threshold *p* < 0.0055). Furthermore, in the AD group, there was no correlation between disease duration, MMSE score, and any of the three markers.

### Correlation of CSF CNPase, MBP, and NG2 with p-Tau and 14-3-3 levels

In the CJD group, no correlation was found between the three CSF markers and the corresponding levels of p-Tau and 14-3-3 protein (*p* > 0.05). Similarly, in the AD group, no significant correlations were observed between the three CSF targets and the corresponding levels of total tau, p-Tau, amyloid-β 1–40, or amyloid-β 1–42 (*p* > 0.05).*i*

### Evaluation of the relationship between CSF levels of Myelin Basic Protein (MBP) and Neurofilament Light Chain (NfL) in the primary cohort and in a second independent cohort

To assess whether elevated CSF MBP levels in CJD might indirectly reflect neuroaxonal degeneration, we quantified CSF NfL concentrations in the discovery cohort in all CJD, AD, and control subjects for whom additional material was available (CJD n = 13/18; AD n = 11/18; CTRL n = 11/18). As expected, NfL values were significantly higher in CJD compared to AD (*p* = 0.003) and controls (*p* < 0.0001), and AD subjects also showed higher values compared to controls (*p* = 0.04). After adjusting for age and NfL, MBP levels remained significantly higher in CJD compared to AD (*p* = 0.029), and NfL did not exert a significant effect in this model (*p* > 0.05), indicating that MBP elevations in this cohort are not explained by neuroaxonal degeneration in either disorder.

In a second independent cohort including CJD and AD cases, MBP and NfL levels were quantified again (study population characteristics can be found in Table 2). NfL concentrations in the AD group were comparable to those observed in the first cohort (cohort 2 median = 1323 pg/ml vs. cohort 1 median = 1596 pg/ml, *p* > 0.05). However, AD patients in the second cohort showed significantly higher tau and p-tau181 levels (total tau: 700 pg/ml vs. 506 pg/ml, *p* = 0.04; p-tau181: 103 pg/ml vs. 73 pg/ml, *p* = 0.03), consistent with more pronounced neuroaxonal involvement. In line with this, AD patients exhibited a substantially more advanced amyloid biomarker profile, reflected by markedly lower Aβ42-values (cohort 1 median = 745 pg/ml, cohort 2 median = 420 pg/ml, *p* = 0.004; Tables 1 and 2). Although amyloid pathology does not directly correspond to neuroaxonal injury, the combination of lower amyloid ratios and higher tau/p-tau values indicates a more advanced disease stage in the second AD cohort. In this cohort MBP levels did not differ between CJD and AD (*p* > 0.05), again applying linear regression ajdusted for NfL, which exerted a strong independent effect in the model (*p* = 0.002). Notably, absolute MBP concentrations were higher in this validation cohort than in the discovery cohort in both groups but AD patients showed a disproportionately stronger increase comapred to CJD (AD median: cohort 1 = 2.8 ng/ml vs. cohort 2 = 10.3 ng/ml, *p* =  < 0.0001; CJD median: cohort 1 = 5.2 ng/ml vs. cohort 2 = 9.9 ng/ml, *p* = 0.0004). This pattern further supports the idea that MBP is increasingly affected by secondary myelin breakdown in later stages of the disease, when neuroaxonal degeneration is more pronounced.

**Table 2:** Characteristics of the study population in the second independent cohort

	CJD	AD
Patients (n)	28	20
Age (years)	71 (45)	76 (20)
Male/female	13/15	9/11
p-Tau pg/ml	NA	103 (217)
Tau pg/ml	NA	700 (1237)
Amyloid-β 1–40 pg/ml	NA	10,805 (15,157)
Amyloid-β 1–42 pg/ml	NA	420 (543)
Amyloid-β 1–42/1–40 ratio	NA	0.04 (0.02)
Neurofilament light pg/ml	5615 (17,272)	1323 (4853)

Data is presented as median (range). CJD = Creutzfeldt–Jakob disease, AD = Alzheimer’s disease, NfL = neurofilament light chain, NA = not applicable

### Stratification for CJD subtypes

Stratification by the CJD subtype (VV2 n = 5, MM/MV1 n = 12, MV2K n = 1) revealed no significant differences in CNPase, MBP, or NG2 levels between subgroups (*p* > 0.05) in the primary cohort. CJD patients from cohort 1 were pooled with those from the second independent cohort and stratified by molecular subtype to increase statistical power for MBP and NfL comparisons (VV2 n = 9, MM/MV1 n = 33, MV2K n = 4). Consistent with the findings from cohort 1, no significant subtype differences in MBP levels were detected (*p* > 0.05, supplemental Fig. 3). In contrast, the VV2 subtype showed significantly higher NfL concentrations compared to MM/MV1 (7877 vs. 4532 pg/ml, *p* = 0.016), and NfL levels in MV2K approached significance when compared to MM/MV1 (7892 vs. 4532 pg/ml, *p* = 0.051).

### Association between CSF CSF CNPase, MBP and NG2 levels and survival in CJD cases

To evaluate the association between survival time and biomarker levels, Spearman correlation analyses were performed. In the primary cohort, no significant correlations were observed between survival and MBP, NG2, or CNPase levels (all *p* > 0.05). In the second cohort, MBP levels again showed no significant association with survival rates (*p* > 0.05).

### Oligodendrocyte quantification in post-morten autopsy tissue

Oligodendrocyte quantification was conducted using post-mortem brain tissue from an independent cohort obtained during routine clinical autopsies (CJD n = 5; control n = 4). A total of 48 ROIs from controls and 60 ROIs from CJD patients were analyzed. The density of p25-positive mature oligodendrocytes was determined. No significant differences were observed in the total number of p25-positive cells between groups, nor in analyses stratified by cortical and white matter regions (*p* > 0.0.5) (Fig. 3A–C). In addition, the brains of patients with CJD were divided into two groups based on their levels of cortical PrP deposits, with the classification being low to moderate or high. These groups were then compared with the control group. Once more, no significant differences were identified (*p* > 0.05) (see Fig. 3D, E).

## Discussion

White matter abnormalities and oligodendrocyte dysfunction have been only sparsely studied in prion diseases. The selection of the three CSF biomarker candidates evaluated in our study (MBP, NG2, CNPase) was based on their potential to reflect different characteristics of oligodendrocyte and myelin maintenance in sporadic Creutzfeldt–Jakob patients in vivo. Additionally, we quantified p25-positive mature oligodendrocytes in post-mortem brain tissue in an independent cohort including patients with sporadic CJD and non-neurodegenerative controls to evaluate if there is a significant loss of oligodendrocytes associated with CJD.

MBP, the second most abundant protein in CNS myelin (around 30%), is a structural core protein localized within the compacted regions of the myelin sheath [18]. Its release into the CSF has been discussed as a marker for demyelination, for example in patients with neuroinflammatory diseases like Multiple Sclerosis [19]. Recently, we reported elevated levels of MBP in the CSF of patients with atypical parkinsonism in comparison to idiopathic Parkinson's disease, potentially suggesting myelin disruption as a part of the underlying disease mechanism [20]. Currently, we observed higher MBP levels in CJD compared to AD and also control subjects (Fig. 1), compliant with histopathological and imaging studies demonstrating disrupted white matter integrity in CJD. In accordance, MRI diffusion imaging studies on CJD reported widespread white matter involvement characterized by reduced mean diffusivity [2]. Furthermore, post-translational modifications of MBP, particularly citrullination, have been implicated in myelin disruption in CJD [4]. Theoretically, elevated MBP levels could result from secondary axonal degradation following neuronal loss. To evaluate the influence of neuroaxonal injury on myelin-basic protein levels, neurofilament light chains were quantified. In the primary cohort, linear regression analysis revealed no association between MBP and NfL levels and the group difference in MBP levels between CJD and AD remained significant even after adjustment for NfL. This indicates that MBP elevations in CJD cannot be explained solely by neuroaxonal degeneration.

To explore whether a more advanced stage of Alzheimer’s disease might alter this relationship, we analysed a second independent cohort. Here, AD patients displayed more advanced biomarker abnormalities, including higher total tau and p-tau181 levels in the CSF and reduced Aβ42 concentrations compared to the primary cohort, consistent with more advanced pathology. Notably, absolute MBP concentrations were higher in this cohort for both AD and CJD compared to the discovery cohort, with AD patients showing a disproportionately stronger increase. In this cohort, MBP levels did not differ between CJD and AD, and NfL showed a strong independent association with MBP. This pattern suggests that, in the later stages of neurodegeneration, MBP is increasingly affected by the secondary breakdown of myelin driven by axonal injury. Taken together, these findings suggest that the relationship between MBP and neuroaxonal degeneration depends on the clinical context. In AD, MBP appears to be more closely linked to neuroaxonal degeneration in patients with more advanced biomarker changes. In CJD, however, MBP levels were elevated in both cohorts, and this increase could not be explained by NfL alone, indicating that myelin or oligodendroglial involvement may represent a primary feature rather than a secondary effect of axonal loss. In line with this, analyses across CJD subtypes showed that MBP levels did not differ between subtypes, even though NfL levels were clearly higher in VV2 and MV2K compared to MM/MV1, consistent with the more pronounced neuroaxonal damage in these subjects.

Despite the absence of routine evaluation of AD biomarkers in the CSF of CJD, we argue against the potential impact of unidentified Alzheimer's disease (AD) co-pathology. Clinically, none of the CJD patients exhibited features suggestive of concomitant Alzheimer's disease, and the distinct neurodegeneration observed in CJD would be expected to overshadow mild AD-type changes.

2′,3′-Cyclic-nucleotide 3′-phosphodiesterase (CNPase) is a myelin-associated enzyme that constitutes around 4% of the total CNS myelin. Its precise enzymatic role remains incompletely understood. Unlike MBP, it is not a structural component of compacted myelin, instead it has been observed in non-compact myelin areas like cytoplasmic domains and in paranodal areas, where it contributes to their structural stabilization. CNPase is predominantly expressed in mature myelinating oligodendrocytes, where it has been implicated in RNA transport, microtubule assembly besides maintaining the cytoskeletal dynamics critical for myelin sheath stability and oligodendrocyte survival [18, 21, 22]. Interestingly, despite a significant increase in CSF CNPase levels in CJD compared to controls, we found no significant difference in oligodendrocyte cell numbers between CJD and controls in post-mortem brain tissue (Fig. 3). We hypothesize that the observed CNPase elevation in CJD is therefore not due to extensive oligodendrocyte loss, but more likely reflects sublethal stress, metabolic dysfunction or impaired vesicular processing in otherwise structurally preserved cells. In line with our findings, transcriptomic analyses of the frontal cortex in CJD brains have reported downregulation of genes involved in oligodendrocyte metabolism and myelin maintenance (e.g., GALC and MCT1) [5]. Even though a relevant proportion of AD and control samples showed values below the LOQ, the high CNPase levels observed in CJD support biological plausibility and cannot be attributed to an assay-related bias. Nevertheless, the frequent sub-LOQ values of the CNPase assay reduced its analytical sensitivity in non-CJD groups. In contrast, MBP concentrations were reliably quantifiable across all diagnostic groups, making MBP the more robust and informative marker in our study.

Neural/glial antigen 2 (NG2) is a transmembrane chondroitin sulfate proteoglycan that is widely recognized as a marker for oligodendrocyte precursor cells (OPCs). NG2-positive OPCs are distributed throughout the central nervous system and contribute to the physiological maintenance and turnover of oligodendrocytes [23]. In pathological states (e.g., traumatic brain injury), NG2-postive OPCs are migrate to sites of damage, where they can differentiate into mature oligodendrocytes to support remyelination [24]. We hypothesize that the absence of NG2 regulation in CJD compared to AD and controls (Fig. 1C) may suggest that the OPC population is not being mobilized, despite evidence of oligodendrocyte dysfunction and ongoing myelin damage as discussed before. We speculate that the glial environment in CJD may be non-permissive for regeneration, possibly because of distinct prion-induced neurotoxicity. On the other hand, unchanged NG2 levels could mean that OPCs are not mobilized because mature cells do not die off to a greater extent. Interestingly, an important role for NG2 glia against prion toxicity has been recently described in brains of prion-inoculated mice. Here, depletion of NG2 glia exacerbated prion-induced neurodegeneration and accelerated prion pathology due to enhanced biosynthesis of prostaglandin E2 by microglia [6].

Our study presents, to the best of our knowledge, the first evaluation of oligodendrocyte- and myelin-associated markers in the cerebrospinal fluid of patients with sporadic Creutzfeldt–Jakob disease. We assume that increased CSF levels of MBP and CNPase, combined with unaltered CSF levels of NG2 and stable oligodendrocyte numbers in post-mortem CJD brains, points at least partially toward myelin damage and oligodendroglial dysfunction as primary mechanisms of white matter pathology in CJD. Due to their discriminative value (Fig. 2), CNPase and MBP may complement existing neuronal biomarkers in CJD.

CSF analysis offers a powerful potential to explore disease mechanisms in vivo but clearly has some limitations. The interpretation of elevated CSF CNPase levels in CJD as a potential indicator of oligodendrocyte dysfunction rather than an indicator of cell loss is supported by postmortem cell counts but remains indirect.. In addition, our study provided only data from a single time point in the disease course, and we are therefore missing information about dynamics of the concentration of the different myelin markers.. A further limitation of this study is the absence of paired CSF and post-mortem brain tissue from the same individuals. As a result, a direct correlation between CSF biomarker levels and oligodendroglial pathology was not feasible, and future multicenter studies may help to address this aspect.

## Conclusions

Our findings indicate that white matter pathology in CJD is not merely a downstream consequence of neuronal degeneration but may be part of the underlying disease mechanism in prion diseases. To date, most of the biomarker research in CJD has focused on neuronal damage (e.g. total tau protein, 14-3-3 protein) and prion protein aggregation (e.g. RT-QuIC assay), while some further studies have also examined astroglial, microglial, inflammatory, and synaptic markers [25–27]. However, only very few investigations have addressed oligodendroglial or myelin-associated CSF markers. The results of this study demonstrate that myelin and/or glia-derived markers capture a distinct and clinically relevant part of the disease process of CJD.

## Supplementary Information

Below is the link to the electronic supplementary material.

Additional file 1. clinical characteristics of the post-mortem cohort.

Additional file 2. Supplementary material containing inclusion criteria, assay metrics and graphical illustration of CJD subtype stratification (MBP values).

## Data Availability

The datasets used and/or analyzed during the current study are available from the corresponding author on reasonable request.

## Abbreviations

AD: Alzheimer's diesease
AUROC: Area under the receiver operating characteristic curve
CJD: Creutzfeldt–Jakob disease
CNPase: Cyclic nucleotide 3′-phosphodiesterase
CSF: Cerebrospinal fluid
CTRL: Controls
MBP: Myelin basic protein
LOQ: Limit of quantification
NG2: Neural/glial antigen 2
RT-QuIC: Real-time quaking-induced conversion
