# Supplementary material for: Alterations in cerebrospinal fluid levels of myelin- and oligodendrocyte-related proteins in sporadic Creutzfeldt–Jakob disease
Source: Acta Neuropathol Commun. 2026 Feb 10;14:51. doi: 10.1186/s40478-026-02247-5 (PMC12930826; doi:10.1186/s40478-026-02247-5)
Supplement: Supplementary file 2 — Additional file 2. Supplementary material containing inclusion criteria, assay metrics and graphical illustration of CJD subtype stratification (MBP values). [file 40478_2026_2247_MOESM2_ESM.docx]

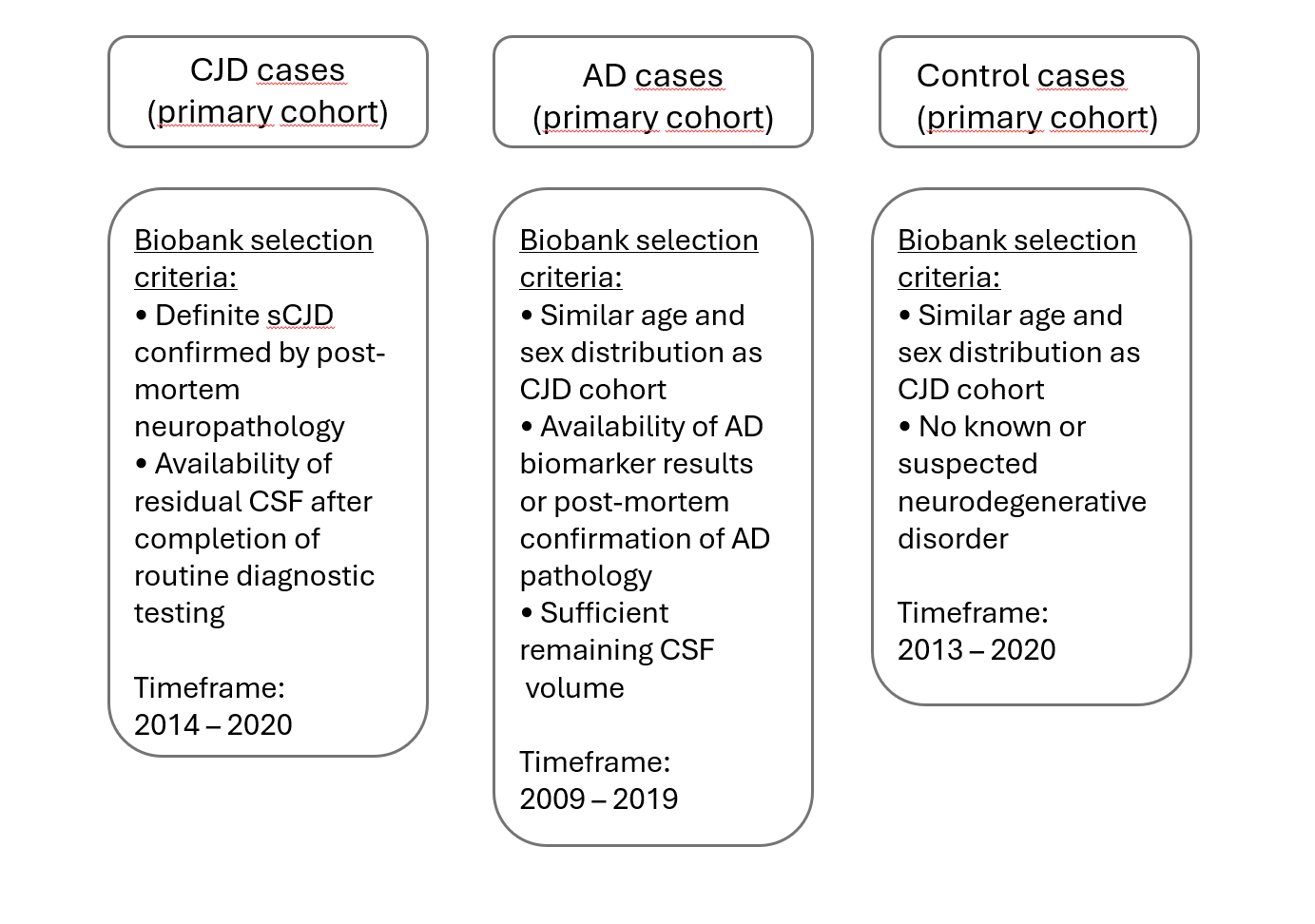


**Supplemental Figure1**: Selection criteria for the first CSF cohort.


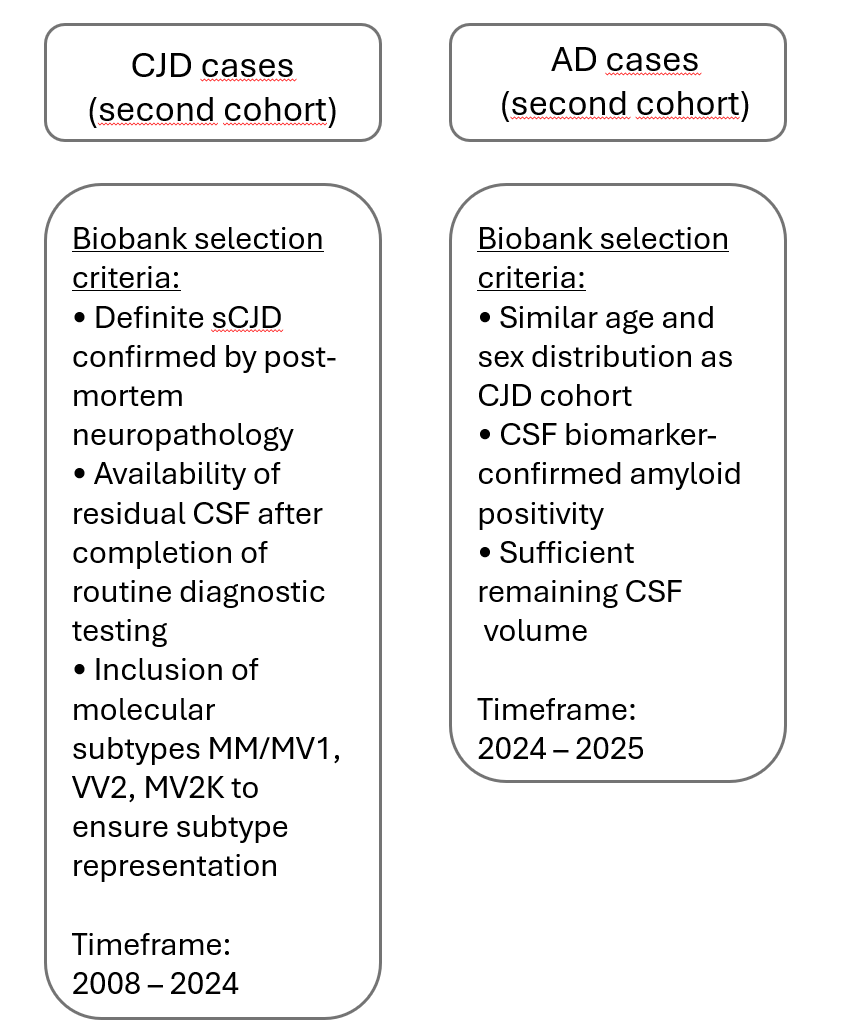


**Supplemental Figure2**: Selection criteria for the second independent CSF cohort.


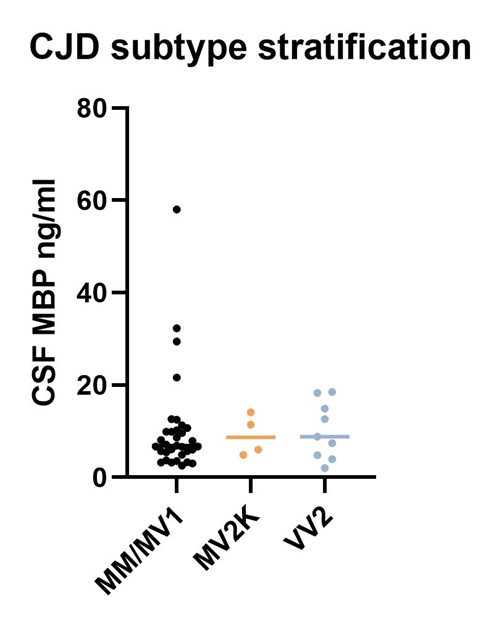


**Supplemental Figure3**: Stratification of CJD subtypes revealed no significant differences concerning MBP levels.

-------------------------------------------------------------------------------------------------------------------------

**Myelin Basic Protein (MBP) ELISA metrics (according to the manufacturer)**

| Detection | HRP-based ELISA, colorimetric detection by dual wavelength absorbance at 450 nm and 630 nm as reference filter |
| --- | --- |
| Dynamic Range | 0.35-10.5 ng/mL |
| Limit of Detection | 0.093 ng/mL |
| Species Reactivity | Enquire about animal-specific cross-reactivity |

Sample Type Cerebrospinal Fluid

Intra-Assay: CV 1.6–5.9 %

Inter-Assay: CV 1.7–3.4 %

**Human NG2 (CSPG4) ELISA metrics (according to the manufacturer)**

Detection method Colorimetric

Sample types Cell culture media, Citrate plasma, Serum

Sensitivity = 25.5 pg/mL

Range 156.25 - 10000 pg/mL

Intra-Assay: CV 3.5%

Inter-Assay: CV 0.5%

**Human CNP (2',3'-cyclic-nucleotide 3'-phosphodiesterase) ELISA metrics (according to the manufacturer)**

Sample Type Serum, Plasma, Cell Culture Supernates, Cell Lysates, Tissue Homogenates, Other Biological Fluids

Detection Range 0.313-20ng/ml

Sensitivity 0.188ng/ml

Intra-Assay: CV<8%

Inter-Assay: CV<10%
